# Liraglutide Improves Endothelial Function via the mTOR Signaling Pathway

**DOI:** 10.1155/2021/2936667

**Published:** 2021-08-16

**Authors:** Han Wu, Cheng Xiao, Yiting Zhao, Hongchao Yin, Miao Yu

**Affiliations:** ^1^Department of Endocrinology, Key Laboratory of Endocrinology, National Health Commission, Peking Union Medical College Hospital, Peking Union Medical College and Chinese Academy of Medical Sciences, Beijing 100730, China; ^2^Department of PET-CT Center, Cancer Hospital, Peking Union Medical College and Chinese Academy of Medical Sciences, Beijing 100021, China; ^3^Department of Pathology, Institute of Basic Medical Sciences, Peking Union Medical College and Chinese Academy of Medical Sciences, Beijing 100730, China

## Abstract

**Background:**

Mammalian target of rapamycin (mTOR) is crucial for endothelial function. This study is aimed at assessing whether the glucagon-like peptide-1 (GLP-1) analogue liraglutide has a protective effect on endothelial function via the mTOR signaling pathway.

**Methods:**

Human umbilical vein endothelial cells (HUVECs) were administered liraglutide (100 nM) for 0, 10, 30, 60, 720, and 1440 minutes, respectively. Then, the expression and phosphorylation levels of mTOR, mTOR-Raptor complex (mTORC1), and mTOR-Rictor complex (mTORC2) were determined by Western blot and immunoprecipitation, while mTORC1 and mTORC2 expression was blocked by siRNA-Raptor and siRNA-Rictor, respectively. Akt phosphorylation was detected by Western blot. HUVECs were then incubated with liraglutide in the absence or presence of Akt inhibitor IV. Nitric oxide (NO) release was assessed by the nitrate reductase method. Phosphorylated endothelial nitric oxide synthase (eNOS), human telomerase reverse transcriptase (hTERT), and apoptosis-related effectors were assessed for protein levels by Western blot. Telomerase activity was evaluated by ELISA.

**Results:**

Sustained mTOR phosphorylation, mTORC2 formation, and mTORC2-dependent Akt phosphorylation were induced by liraglutide. In addition, eNOS phosphorylation, NO production, nuclear hTERT accumulation, and nuclear telomerase activity were enhanced by mTORC2-mediated Akt activation. Liraglutide also showed an antiapoptotic effect by upregulating antiapoptotic proteins and downregulating proapoptotic proteins in an mTORC2-Akt activation-dependent manner.

**Conclusion:**

Liraglutide significantly improves endothelial function, at least partially via the mTORC2/Akt signaling pathway.

## 1. Introduction

Accelerated atherosclerosis characterizes type 2 diabetes (T2DM). Endothelial cell dysfunction (ECD) in premature atherosclerosis found in T2DM is considered the causal factor in atherogenesis. ECD is characterized by decreased endothelial nitric oxide synthase (eNOS) activity and nitric oxide (NO) release, reduced telomerase activity, and elevated cytokine expression, resulting in impaired vasodilation and increased sensitivity to apoptotic stimuli, which could worsen the deficits and promote the pathogenesis and progression of atherosclerosis. Therefore, exploring the potential mechanism and identifying a strategy for the improvement of endothelial cell function are critical for preventing and treating macrovascular complications in T2DM.

Liraglutide represents a glucagon-like peptide-1 (GLP-1) analogue employed in T2DM treatment. Similar to endogenous GLP-1, liraglutide induces postprandial insulin secretion, reduces glucagon release, and enhances satiety. Mounting evidence suggests GLP-1 analogues might equally decrease the risk of cardiovascular diseases in individuals with T2DM [1, 2]. In addition to its well-documented incretin role, GLP-1 affects the cardiovascular system by direct action on the endothelium, in which specific receptors for GLP-1 have been detected [3]. In particular, GLP-1 induces vasodilatation and improves endothelial function [4]. However, the mechanism underlying these effects remains largely unknown.

Previous findings have demonstrated that the mammalian target of rapamycin (mTOR) is closely associated with endothelial cell function. Recent studies suggested GLP-1 has protective effects on pancreatic beta cells [5], cardiomyocytes [6], and neuronal cells [7], including antiapoptotic and antioxidative activities via the mTOR signaling pathway. However, studies assessing the associations of GLP-1's beneficial effects on endothelial cell function with mTOR/Akt signaling are scarce.

The purpose of the present work was to assess whether the protective effects of the GLP-1 analogue liraglutide on endothelial function involve the mTOR signaling pathway.

## 2. Materials and Methods

### 2.1. Cell Culture

Human umbilical vein endothelial cell (HUVEC, ScienCell, America) isolation was performed from human umbilical cord vein specimens by collagenase treatment as previously described [8]. HUVECs were cultured in ECM medium (ScienCell) at 37°C in a humid environment with 5% CO_2_ and used at passages 3–5.

### 2.2. Immunoblot and Immunoprecipitation

These assays were carried out according to previous descriptions [9].

### 2.3. Transfection of siRNAs into HUVECs

For gene silencing, mTOR, Raptor, Rictor, and control siRNAs, respectively, were transfected into HUVECs following a previous report [10]. Briefly, HUVECs (2 × 10^5^ cells per well of 6-well plates) were cultured in ECM medium (2 ml) to 60% confluency. This was followed by 7 h of incubation in the presence of respective siRNAs. After transfection, HUVECs were further cultured for 24 h. Finally, mTOR, Raptor, and Rictor expression levels were analyzed by Western blot.

### 2.4. Telomerase Assay

Cells were seeded into 6-well plates (2 × 10^5^ cells per well) and incubated with liraglutide in the absence or presence of Akt inhibitor IV. Following PBS washes, the HUVECs were lysed as previously described [11]. Protein amounts in the collected supernatants were determined by the Bradford method. Telomerase activity was assessed using 3 *μ*g of protein with a TeloTAGGG Telomerase PCR ELISA Plus Kit (Roche).

### 2.5. NO Measurement

NO release from HUVECs was assessed with a NO assay kit (AO12, Nanjing Jiancheng Bioengineering Institute) by the nitrate reductase method.

### 2.6. Statistical Analysis

Data are mean ± SEM from 3 or more experiments performed independently. Student's *t*-test was employed to compare group pairs. One-way analysis of variance (ANOVA) with subsequent Tukey post hoc test was employed for multiple group comparisons. *p* < 0.05 indicated statistical significance.

## 3. Results

### 3.1. Liraglutide Increases mTOR Phosphorylation in HUVECs

**Figure 1:**
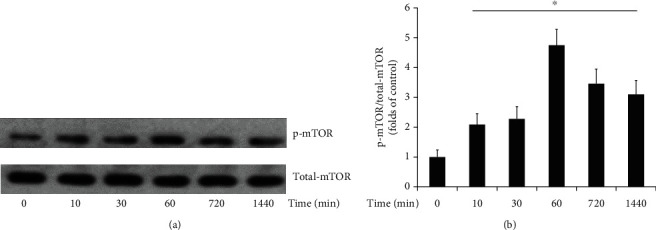
HUVECs were treated with liraglutide (100 nM) for 0, 10, 30, 60, 720, and 1440 minutes, respectively. The expression and phosphorylation of mTOR were detected by Western blot (a). Ser2448 phosphorylation levels relative to total mTOR protein amounts were assessed by densitometric quantification. Data are mean ± SEM (*n* = 5). ^∗^*p* < 0.05 vs. 0-minute incubation (b).

HUVECs were administered liraglutide (100 nM) for 0, 10, 30, 60, 720, and 1440 minutes, respectively, and immunoblot was carried out to assess the expression and phosphorylation of mTOR (Figure 1(a)). As shown in Figure 1(b), liraglutide significantly increased mTOR phosphorylation; Ser2448 phosphorylation in mTOR increased from 10 min and peaked at 60 min, with elevated amounts remaining until the 1440 min time point.

### 3.2. Liraglutide Induces mTORC2-Dependent Akt-Ser473 Phosphorylation

**Figure 2:**
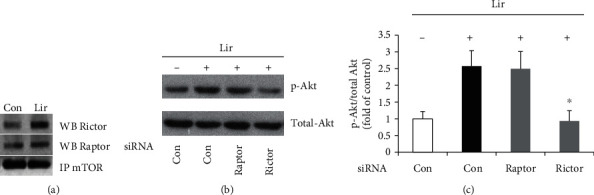
HUVECs were administered liraglutide at 100 nM for 60 min. Immunoprecipitation (IP) was performed with anti-mTOR antibodies; Western blot (WB) with anti-Rictor and anti-Raptor antibodies was next carried out to assess immunoprecipitated complexes (a). HUVECs were administered liraglutide at 100 nM for 60 min in three groups, with a vehicle control group assessed in parallel. The vehicle group was transfected with control siRNA, while HUVECs treated with liraglutide were transfected with control siRNA, Rictor-specific siRNAs, and Raptor-specific siRNAs, respectively. Akt phosphorylation at Ser473 and total Akt levels were detected by Western blot (b). Akt phosphorylation levels relative to total Akt protein amounts were assessed by densitometric quantification. Data are mean ± SEM (*n* = 5). ^∗^*p* < 0.05 vs. HUVECs transfected with control siRNA and treated with liraglutide (c).

GLP-1 activates protein kinase B (Akt), which activates eNOS via direct phosphorylation. Therefore, the effects of liraglutide on activated phosphor-Akt expression were assessed. Akt phosphorylation at Ser473 (primary phosphorylation site) was increased in HUVECs treated with liraglutide for 60 min, whereas total Akt levels remained unchanged (Figure 2(b)). It is known that mTOR contributes to two different multiprotein complexes: mTORC1, a demonstrated target of rapamycin, comprises mTOR, Mlst8, and Raptor; mTORC2, initially thought to be rapamycin-insensitive, comprises mTOR, Mlst8, and Rictor. Next, mTORC1 and mTORC2 involvement in the protective effects of liraglutide on HUVEC function was assessed. Previous findings have demonstrated that mTORC2 phosphorylates Akt at Ser473. To assess whether liraglutide promotes mTORC2 synthesis in HUVECs, coexpression and association of mTOR with Rictor were evaluated after treatment with liraglutide (Figure 2(a)). Immunoblot showed Rictor and Raptor protein amounts were invariably decreased in HUVECs transfected with Rictor- and Raptor-specific siRNAs, separately. No effects were observed in the control siRNA group. Next, siRNA-transfected HUVECs were administered liraglutide for 60 min. The results showed that siRNA-associated Rictor knockdown markedly reduced liraglutide-dependent Akt phosphorylation in comparison with the control siRNA group, while siRNA-induced Raptor knockdown showed no effects (Figures 2(b) and 2(c)). Taken together, these findings suggested that liraglutide promoted mTORC2 formation and the activation of downstream Akt in HUVECs.

### 3.3. Liraglutide Increases NO Production in HUVECs

**Figure 3:**
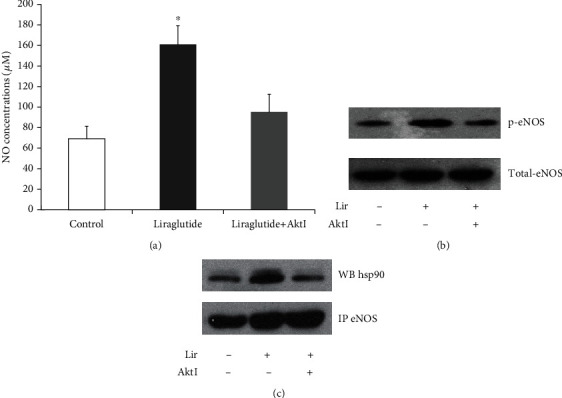
HUVECs were administered liraglutide at 100 nM in the absence or presence of Akt inhibitor IV for 60 min. NO release from HUVECs was assayed by the nitrate reductase method. Data are mean ± SEM (*n* = 3). ^∗^*p* < 0.05 vs. the control group (a). Phosphorylated eNOS was detected by Western blot (b). Immunoprecipitation (IP) was performed with anti-eNOS antibodies, followed by Western blot (WB) with anti-hsp90 antibodies for assessing immunoprecipitated complexes (c).

Endogenous NO has a critical function in endothelial cells. Therefore, we assessed the effects of liraglutide on NO output and the activity of eNOS, the main NO synthetase in endothelial cells. Treatment of HUVECs with liraglutide at 100 nM for 60 min resulted in significantly increased amounts of bioactive NO in cell supernatants compared with control values (Figure 3(a)). In addition, the levels of phosphorylated eNOS (Figure 3(b)) and the e-NOS-hsp90 complex (Figure 3(c)) were also increased after administration of liraglutide, as detected by Western blot and in coimmunoprecipitation experiments, respectively. Since Akt activates eNOS by direct phosphorylation, these effects were blunted by an Akt inhibitor.

### 3.4. Liraglutide Increases the Nuclear Translocation of hTERT and Telomerase Activity in HUVECs via mTORC2/Akt Signaling

**Figure 4:**
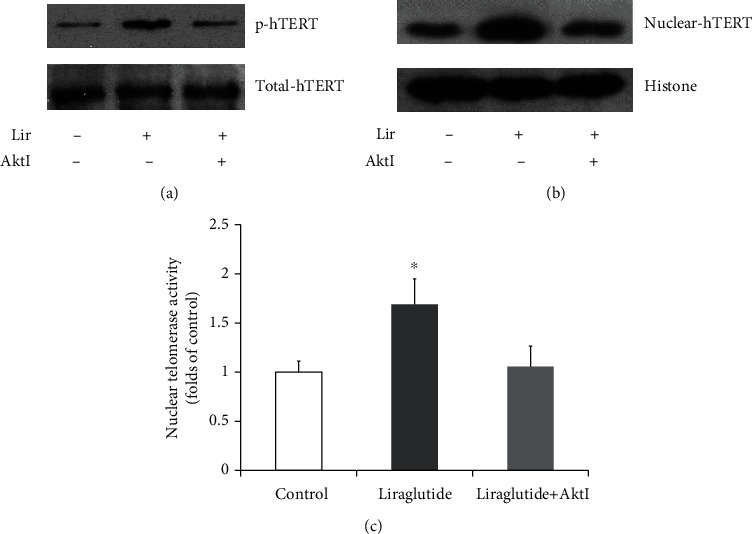
HUVECs were administered liraglutide at 100 nM in the absence or presence of Akt inhibitor IV for 60 min. Phosphorylation of hTERT was assessed by Western blot (a). The expression levels of nuclear hTERT were evaluated by Western blot (b). Nuclear telomerase activity in HUVECs was assessed by ELISA. Data are mean ± SEM (*n* = 3). ^∗^*p* < 0.05 vs. the control group (c).

Cell senescence is controlled by telomerase activity modulated at the hTERT expression, phosphorylation, and nuclear translocation levels. To explore mechanisms downstream the mTORC2/Akt pathway, whether liraglutide regulates hTERT protein expression in HUVECs was assessed by Western blot. As shown in Figure 4, p-hTERT levels (Figure 4(a)) and nuclear accumulation of hTERT (Figure 4(b)) as well as nuclear telomerase activity (Figure 4(c)) in HUVECs were increased upon treatment with liraglutide. Such effects were blunted by Akt inhibitor IV.

### 3.5. Effects of Liraglutide on Apoptosis-Associated Protein Expression Levels in HUVECs

**Figure 5:**
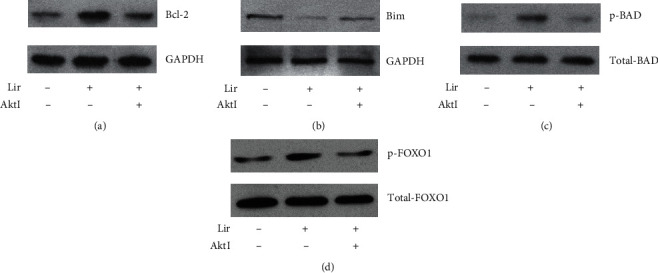
HUVECs were administered liraglutide at 100 nM in the absence or presence of Akt inhibitor IV for 60 min. The (a, b) protein expression levels of Bcl-2 and Bim and (c, d) phosphorylation levels of BAD and FOXO1 were detected by Western blot.

Apoptotic-related proteins, including Akt, B cell lymphoma- (Bcl-) 2, and Bcl-2-associated death promoter (BAD), were quantitated by Western blot (Figures 5(a)–5(d)).

Liraglutide promoted Akt phosphorylation at Ser473 with a subsequent increase of Bcl-2 protein expression levels (Figure 5(a)); meanwhile, enhanced BAD phosphorylation (Figure 5(c)) was observed in GLP-1-treated HUVECs. In addition, decreased Bim (proapoptotic protein) levels (Figure 5(b)) were obtained via phosphor-FOXO1 (Figure 5(d)) after liraglutide administration. Interestingly, these liraglutide effects were overturned upon administration of Akt inhibitor IV. These results indicated that liraglutide could attenuate apoptosis in HUVECs by regulating apoptosis-associated proteins via the mTOR/Akt pathway.

## 4. Discussion

This work showed liraglutide protected endothelial function via the mTOR signaling pathway by inducing mTOR phosphorylation, mTOR-Rictor complex formation, and mTORC2-dependent Akt phosphorylation. In addition, mTORC2-associated Akt activation enhanced the downstream NO production, hTERT translocation into the nucleus, and nuclear telomerase activity, upregulating and inhibiting antiapoptosis and proapoptosis proteins, respectively, in HUVECs. The above findings provide critical mechanistic insights into the effects of liraglutide on HUVECs.

The mTOR protein regulates cell growth, proliferation, viability, and functions [12]. We observed that liraglutide potently protected HUVECs through a mechanism involving mTOR activation. It is known that mTOR activity is modulated through phosphorylation. As shown above, phosphorylation levels of mTOR at Ser2448 increased from 10 min and peaked at 60 min, remaining high until 1440 min following treatment of liraglutide in HUVECs. The mTOR protein represents a component of two different multiprotein complexes, including mTORC1 and mTORC2. mTORC2 phosphorylation promotes mTOR and Akt phosphorylation, enhancing cytoskeletal actin microfilament organization [13]. We found that mTORC2 formation was increased after treatment with liraglutide in HUVECs; meanwhile, its knockdown both reduced liraglutide-associated Akt phosphorylation and blunted the protective effect of liraglutide. Therefore, long-term administration of liraglutide could promote Akt activation by increasing phosphorylated mTOR amounts and mTORC2 levels in HUVECs, indicating that liraglutide protects HUVEC function via the mTORC2/Akt pathway.

NO represents the main endothelial vasodilator, with powerful antiatherosclerotic effects due to its antioxidative, anti-inflammatory, and anticoagulatory properties [14, 15]. Pathological alterations, including insulin resistance and metabolic disorders in T2DM, cause eNOS dysfunction and reduce NO synthesis, which are currently considered the main mechanisms underlying the macrovascular complications of diabetes. Furthermore, GLP-1 increases NO amounts in the coronary effluent from the mouse heart, a vasorelaxation effect blunted by eNOS inhibitors [16], indicating that GLP-1 activates eNOS. Phosphorylation of eNOS at Ser1177 is indispensable for its activation. Consistent with previous findings, this study demonstrated that liraglutide induced NO production via eNOS phosphorylation. Meanwhile, many kinases phosphorylate eNOS, including PKA, Akt, and AMPK, which have significant functions in GLP-1 signaling [17]. This study also demonstrated that Akt inhibition resulted in a reduction of GLP-1-associated NO production, suggesting that GLP-1's vasodilation effects are, at least partly, controlled by the above pathways. These findings corroborate previously reported data showing that GLP-1 induces the proliferation of HUVECs under the control of Akt and eNOS.

In addition to regulating eNOS phosphorylation and endothelial NO production, Akt also controls multiple cell cycle modulators and cell survival pathways. We assessed whether liraglutide inhibits HUVEC senescence. As shown above, liraglutide increased nuclear hTERT accumulation, markedly enhancing nuclear telomerase activity in HUVECs. Such effects were blunted by Akt inhibitor IV and Rictor knockdown, indicating that liraglutide enhances nuclear telomerase activity in HUVECs via mTORC2 and Akt activation.

Multiple reports have demonstrated GLP-1 is antiapoptotic in various cell types, e.g., pancreatic *β* cells, cholangiocytes, neurons, cardiomyocytes, and vascular endothelial cells [18]. It is well accepted that Bcl-2 family members represent central regulators of cell death. For instance, BAD binds to the antiapoptotic protein Bcl-2 or Bcl-Xl to generate a complex that induces apoptosis. However, BAD phosphorylation at Ser136 by Akt causes its release from the above complex; it then forms another complex with cytosolic 14-3-3 proteins, thereby losing its proapoptotic activity [19]. BAD is considered a downstream target of Akt in enhancing cell survival. Previous findings indicate that GLP-1 induces Bcl-2 upregulation, BAD inactivation, and caspase-3 activity reduction in pancreatic *β* cells [20]. Furthermore, GLP-1 increases the Bcl-2 to Bax ratio, reduces cytoplasmic cytochrome c levels, and decreases caspase-9 and caspase-3 activities in HUVECs administered advanced glycation end products (AGEs) [21]. Consistent with these reports, activated mTOR subsequently phosphorylated its downstream effector Akt and increased Bcl-2 expression and BAD phosphorylation, while downregulating the proapoptotic protein Bim and allowing HUVECs to withstand apoptotic stimuli.

The mTOR signaling pathway is closely related to the regulation of cell metabolism. Therefore, we will further study the effects of liraglutide on cell metabolism regulation and mitochondrial function under pathological conditions (such as high-glucose and high-fat condition) and whether it regulates cell homeostasis through autophagy. That will be beneficial to further clarify the cardiovascular protective mechanism of liraglutide. In summary, liraglutide stimulates mTOR phosphorylation, mTORC2 formation, and Akt activation in HUVECs, with protective effects via mTORC2/Akt-dependent increase of nuclear telomerase activity and NO biosynthesis. The current findings reveal new mechanisms underlying liraglutide-associated protection of HUVEC function and suggest mTORC2 to be a critical modulator of HUVEC function.

## Data Availability

The raw data supporting the conclusions of this article will be made available by the authors, without undue reservation.
